# The Role of Serial Imaging in Neurocysticercosis for Disease Resolution

**DOI:** 10.7759/cureus.16790

**Published:** 2021-07-31

**Authors:** Sheyla Gonzalez, Richard Medina-Perez, Danay Herrera, Jose Mario Acosta Rullan, Jose L Batista

**Affiliations:** 1 Internal Medicine, Aventura Hospital and Medical Center, Aventura, USA; 2 Osteopathic Medicine, Nova Southeastern University Dr. Kiran C. Patel College of Osteopathic Medicine, Fort Lauderdale, USA

**Keywords:** generalized tonic clonic seizures, neurocysticercosis, parasitic infection, ring enhancing lesion, taenia solium

## Abstract

Neurocysticercosis (NCC), the most common parasitic infection of the CNS in humans, is a frequent cause of seizure disorders and epilepsy. The cystic larvae *Taenia solium* is endemic to developing countries where the population raises pigs as a reliable source of food, however, massive immigration has now forced the surge of the disease in developed areas making it a worldwide problem. Clinical presentation is affected by the size, number, and location of the lesions within the brain, with the most common manifestations being seizures, headaches, and increased intracranial pressure. The appearance of NCC on radiological imaging helps determine the stage of the disease, required for appropriate antiparasitic treatment. In this article, we detail the case of a patient who presented for recurrent seizures after reportedly undergoing treatment for NCC years prior.

## Introduction

Cysticercosis is a parasitic infection caused by the larvae of the pork tapeworm Taenia solium [1]. The parasite is endemic to developing countries such as Mexico, Central and South America, parts of Africa, Asia, and India [2]. Its prevalence, however, has increased through the years in developed countries due to massive immigration, making it a worldwide disease. 

The tapeworm transmission occurs by fecal-oral contamination of the eggs shed in the stool of human carriers [3]. Pigs become infected by ingesting the worm, contaminated food, or through human feces. Humans subsequently can become infected after ingesting undercooked pork meat containing cysticerci in their muscle tissue or frequently by transmission from household asymptomatic carriers [4]. After ingestion, the embryos hatch in the small intestine and disseminate hematogenously to different parts of the body including the CNS. Over a period of weeks in the tissue, cysticerci develop, forming a membranous wall with fluid and invaginated scolex, characteristically known as cysts [5]. When found in the brain, these cysts are known as neurocysticercosis (NCC).

This parasitic infection is one of the leading causes of acquired and preventable seizures worldwide [6]. Those affected may experience an asymptomatic period that can take as long as three and a half years [7].

## Case presentation

The patient is a 21-year-old female from Peru with a prior history of remote seizures who presented to the hospital via emergency medical services (EMS) after a convulsive episode witnessed at home. As per the family, the patient was speaking when she suddenly started trembling, drooling, and her jaw clenched. The episode lasted approximately 10 minutes and resolved without intervention. A few minutes thereafter, the patient experienced a second tonic-clonic seizure at which time, EMS was called and benzodiazepine was administered as abortive therapy.

Upon further questioning, the patient reported that at age 13 while living in Peru, she experienced similar episodes of seizures for which she underwent brain imaging. At the time, the patient was informed she had an unspecified infection and was treated with a 14-day course of mebendazole. The patient was lost to follow up thereafter as she did not experience further episodes, however; she did endorse frequent headaches and occasional photophobia throughout the years. The patient denied a family history of seizures. 

**Figure 1:**
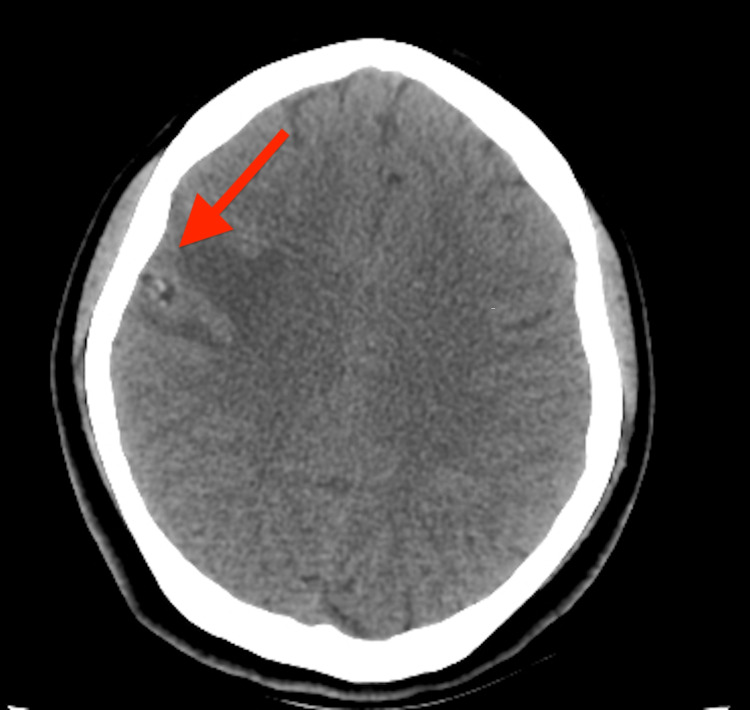
Axial view CT brain showing ring-like hyperdensity with central calcification (red arrow).

**Figure 2:**
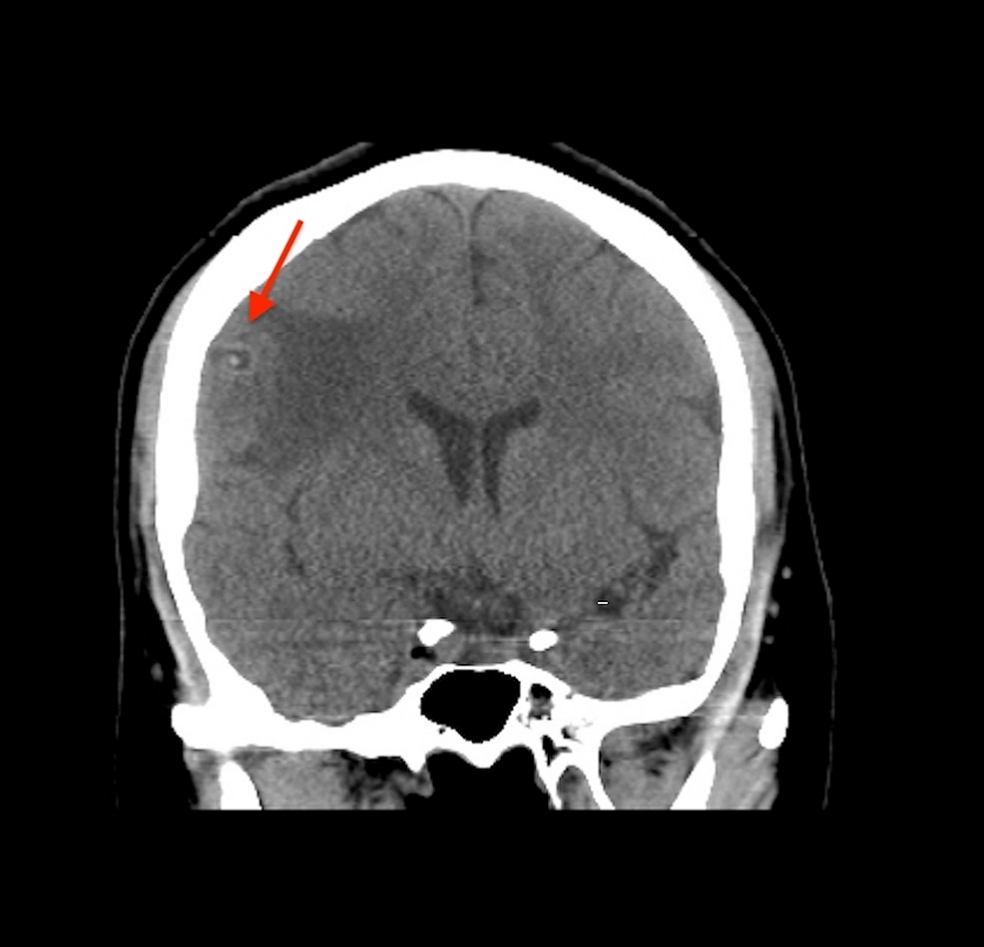
Coronal view CT brain showing ring-like hyperdensity (red arrow) with surrounding vasogenic edema.

Physical exam at presentation was significant for a left-sided facial droop with associated facial numbness, but no further focal neurological deficits were noted. Initial lab work was unremarkable, and the patient remained afebrile and hemodynamically stable. Brain CT showed ring-like hyperdensity with central calcification and vasogenic edema in the right frontal lobe (Figures 1-2).

Presenting patient was started on antiepileptic medication and the neurology team, along with the infectious disease (ID) team, were consulted. A 40 minutes EEG did not show any obvious destabilization, overt epileptiform activity, or hemispheric asymmetry. Given the fact that the patient had vasogenic edema and residual left-sided facial droop, steroid therapy was initiated as per neurosurgery recommendations. 

**Figure 3:**
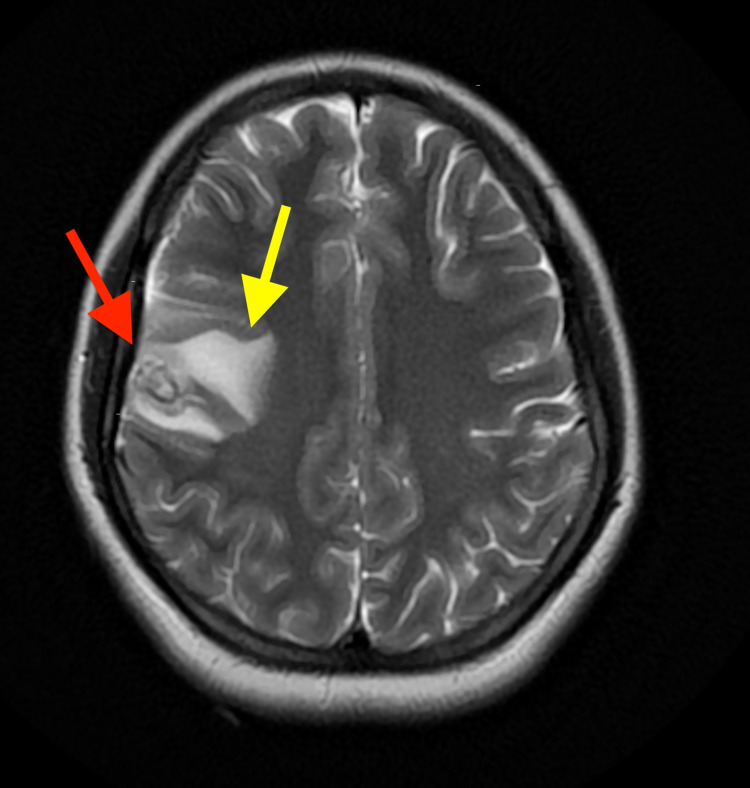
Axial view MRI brain showing solitary cystic mass (red arrow), with surrounding vasogenic edema (yellow arrow).

**Figure 4:**
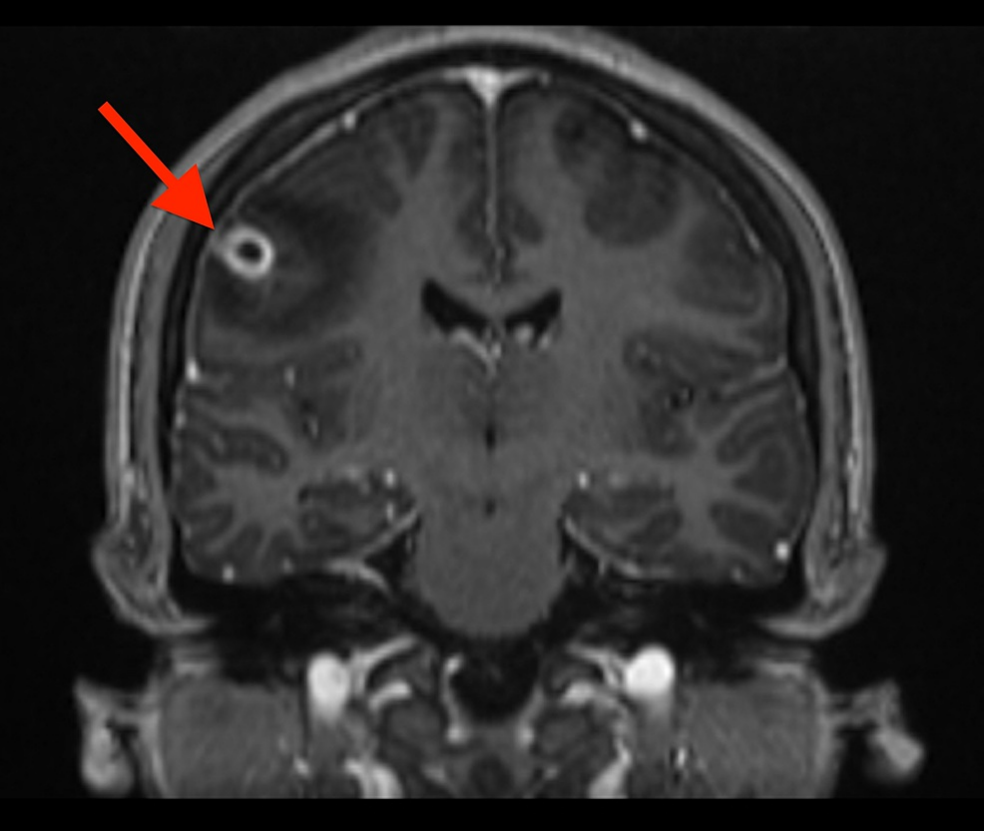
Coronal view MRI brain showing cystic mass with thickened peripheral enhancement (red arrow).

Brain MRI showed a solitary cystic mass with thickened, peripheral enhancement in the right frontal lobe measuring 1.8 x 1.4 x 1.3 cm with surrounding vasogenic edema (Figures 3-4). 

Antibodies testing for T. solium were positive and the patient was diagnosed with NCC colloidal vesicular stage 2 and was started on albendazole 600 mg twice a day (BID) to complete a total of 28 days with concomitant use of a slow steroid taper to be finished a week after the antiparasitic treatment. Three days after admission, the facial droop had resolved, and the patient had not had any more episodes of seizures while on antiepileptic medications. The patient was discharged home a few days later with strict seizures precautions and close follow-up with neurology and ID.

**Figure 5:**
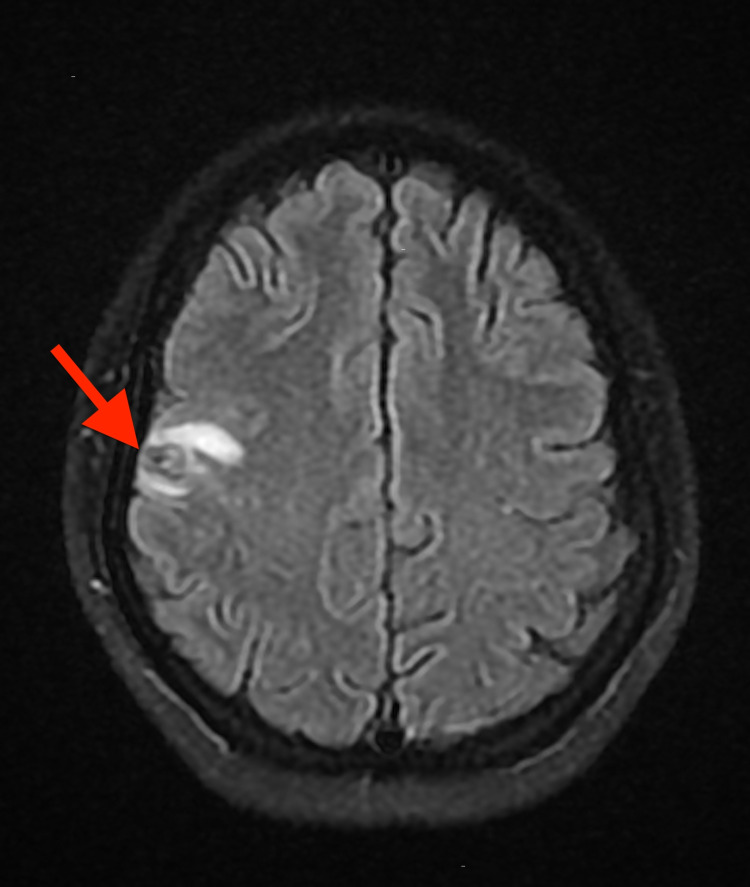
Axial brain MRI showing decrease in size of cystic mass (red arrow).

The patient continued therapy outpatient without complication. MRI was repeated one month after the initiation of treatment, showing an interval decrease in the size of a cystic mass in the right frontal lobe and decreased surrounding vasogenic edema (Figure 5).

**Figure 6:**
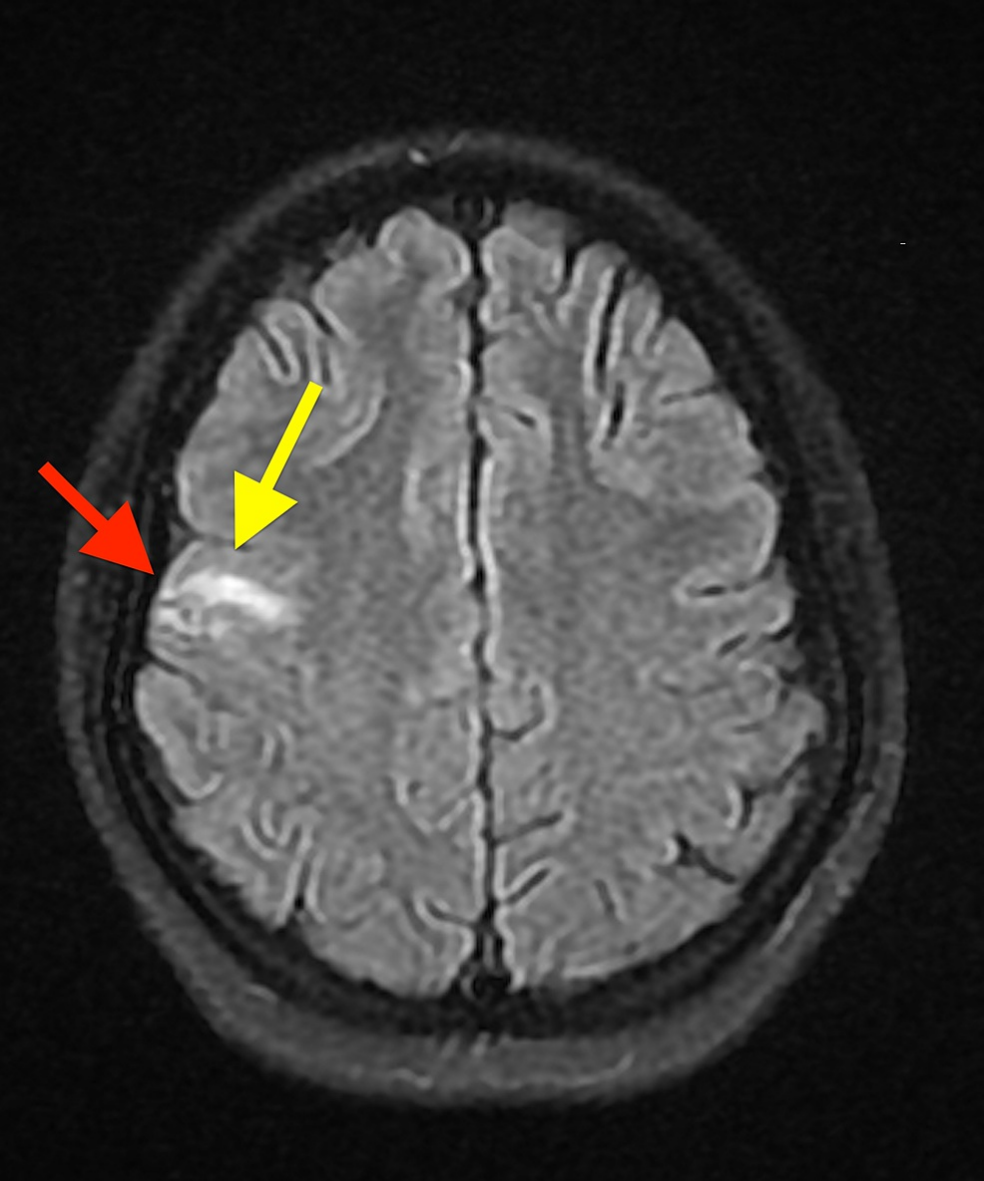
Axial view brain MRI showing peripheral enhancing lesion with an unchanged size (red arrow) and with decreased size of vasogenic edema (yellow arrow).

Two months after treatment, MRI showed an unchanged size of the cystic lesion with decreased size of vasogenic edema versus gliosis (Figure 6).

**Figure 7:**
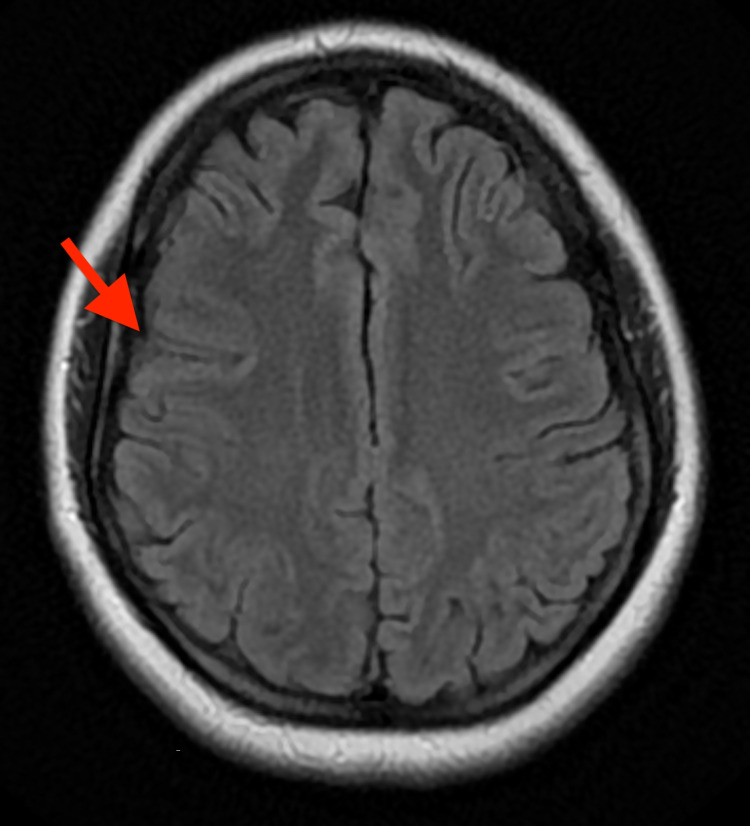
Axial view brain MRI showing a decrease in size of right frontal ring-enhancing lesion (red arrow).

Eight months after treatment completion, the patient continued to be symptom-free and MRI showed significant size reduction of cystic lesion and resolution of surrounding vasogenic edema (Figure 7).

## Discussion

Global data of NCC prevalence is limited and is more commonly seen in regions of Central America, South America, South Africa, Asia, and India. In the United States of America, the majority of cases are seen due to the influx of immigrants from these developing countries [8]. The prevalence of NCC is higher in endemic regions where sanitary conditions are suboptimal. 

Clinical presentation of NCC can vary, symptomatic presentation can include but is not limited to seizures/epilepsy (39%), severe headaches (38%), focal deficits (16%), and signs of increased intracranial pressure (12%) [9]. A high index of suspicion for NCC is paramount for the diagnosis, especially in those with epidemiologic exposure, and is established based on clinical manifestations and relevant imaging showing cystic lesions/calcifications. 

Primary diagnostic tools to evaluate patients with suspected NCC are CT scans and MRI of the brain [10]. These modalities are helpful to detect calcifications, small lesions, surrounding edema, parenchymal cysticerci, and evaluate degenerative changes. Serologic testing with enzyme-linked immuno-transfer blot should be performed for confirmatory evaluation in patients with suspected cysticercosis [10]. A negative serologic test does not exclude the diagnosis of NCC, especially in those with high suspicion as sensitivity is poor with a single parenchymal lesion or calcifications [11]. 

Initial medical management for NCC should be guided directly towards presenting symptoms. Treatment should therefore include anti-seizure and anti-inflammatory therapy, such as corticosteroids, in the setting of convulsive episodes and symptoms secondary to elevated intracranial pressure. In the case of hydrocephalus, either an obstructive or communication surgical approach may be warranted.

Antiparasitic therapy is considered for patients with no signs of elevated intracranial pressure or once this has resolved [12]. In most cases, the duration of antiparasitic therapy ranges from 10 to 14 days except in those with a subarachnoid disease which may warrant a longer treatment course. Once initial management is established, the patient should be closely monitored with repeat imaging every six months until resolution of the cystic lesion is achieved. If no resolution is achieved, repeated antiparasitic therapy may be required.

No vaccine has been studied in humans at this time. A recombinant vaccine has been studied in pigs which has been highly beneficial to reduce cysticercosis in endemic areas [13]. Prevention, therefore, focuses more on education regarding the route of transmission and proper handwashing techniques when handling food.

## Conclusions

NCC is a zoonotic disease that can be prevented by performing appropriate handwashing techniques and by avoiding eating undercooked meat. Since clinical presentation varies, neuroimaging plays a key role in diagnosing and disease resolution. If a resolution is not achieved after initial treatment, a longer course of antiparasitic might be indicated to prevent relapse of the disease.
